# Participant Support for Changes to the Supplemental Nutrition Assistance Program

**DOI:** 10.1001/jamahealthforum.2024.4090

**Published:** 2024-12-06

**Authors:** C. Ross Hatton, Julia A. Wolfson, Alessandra Uriarte, Cindy W. Leung

**Affiliations:** 1 Department of Health Policy and Management, Johns Hopkins Bloomberg School of Public Health, Baltimore, Maryland; 2 Department of International Health, Johns Hopkins Bloomberg School of Public Health, Baltimore, Maryland; 3 Department of Nutrition, Harvard T.H. Chan School of Public Health, Boston, Massachusetts

## Abstract

This cross-sectional study reports participant attitudes regarding 11 Supplemental Nutrition Assistance Program policies and stratified by self-reported political affiliation.

## Introduction

The Supplemental Nutrition Assistance Program (SNAP) helps millions of US families afford food each year. In 2024, policymakers proposed several changes to SNAP, including making unhealthy items ineligible for purchase, limiting the US Department of Agriculture’s ability to increase benefits, and allowing hot and prepared items to be purchased.^1,2^ Although not yet enacted, these changes remain a priority for many policymakers during ongoing Farm Bill discussions. To better understand SNAP participant support for policies that could materially alter the size and use of benefits, we surveyed a nationally representative sample of recent participants about their attitudes toward 11 policies.

## Methods

SNAP Experiences 2024 surveyed SNAP participants from June 7 to 17, 2024, using the probability-based NORC AmeriSpeak panel, with a 22.3% recruitment rate (eAppendix 1 in Supplement 1 provides survey administration details). Respondents provided demographic information and answered questions about 11 SNAP policies, which were selected based on prior research evaluating policy perspectives of SNAP participants.^3^ The survey was fielded among US adults with incomes below 250% of the federal poverty level who participated in SNAP for at least 3 months in 2023. Participants with missing policy support or political party were excluded, resulting in a sample size of 1656. The Harvard T.H. Chan School of Public Health Institutional Review Board (IRB) approved this cross-sectional study. NORC’s IRB obtained written informed consent from all AmeriSpeak panel participants. We followed the STROBE reporting guideline.

We calculated the weighted proportion of respondents who supported each policy, with responses of somewhat support, support, and strongly support dichotomized as support (Appendix 2 in Supplement 1 for question wording). We used χ^2^ tests to compare support by political party, with leaners included with Democrats or Republicans, resulting in 3 categories: Democrat, Independent or none, and Republican. Survey weights were used to ensure representativeness of the sampled population. We conducted all analyses using Stata 18 (StataCorp LLC).

## Results

A total of 1656 adults (55.6% females, 44.4% males; mean [SD] age, 45.5 [17.2] years) completed the survey. Of these respondents, 46.7% identified their political party as Democrat; 26.5% as Republican; and 26.8% as Independent, do not know, or none (Table 1).

**Table 1.** Unweighted and Weighted Sample Characteristics

Characteristic	Unweighted, No. (%)	Weighted, %
ESS	1656	1634
Sex		
Male	459 (27.7)	44.4
Female	1197 (72.3)	55.6
Age, y		
18-29	168 (10.1)	22.9
30-44	553 (33.4)	29.2
45-59	446 (26.9)	20.1
≥60	489 (29.5)	27.8
Race and ethnicity^a^		
Black, non-Hispanic	417 (25.2)	23.0
Hispanic	289 (17.5)	27.3
White, non-Hispanic	822 (49.6)	39.2
Other, non-Hispanic^b^	128 (7.7)	10.6
Educational level		
≤High school diploma	619 (37.4)	61.8
Some college or associate’s degree	746 (45.0)	28.2
≥Bachelor’s degree	291 (17.6)	10.0
Marital status		
Married	449 (27.1)	26.7
Widowed, divorced, or separated	547 (33.0)	27.6
Never married	660 (39.9)	45.7
Household income, $		
<10 000	291 (17.6)	18.4
10 000-19 999	426 (25.7)	25.8
20 000-24 999	173 (10.4)	12.0
25 000-34 999	304 (18.4)	17.6
35 000-49 999	213 (12.9)	11.5
50 000-99 999	249 (15.0)	14.6
Employment status		
Employed	749 (45.2)	45.1
Laid off or looking for work	160 (9.7)	12.2
Not working (retired, disabled, or other)	747 (45.1)	42.7
Metropolitan area residency		
Not in a metropolitan area	281 (17.0)	15.4
In a metropolitan area	1375 (83.0)	84.6
Housing ownership		
Owned or being bought	631 (38.1)	39.6
Rented	934 (56.4)	55.2
Occupied without payment of rent	91 (5.5)	5.2
Region		
Northeast	229 (13.8)	18.8
Midwest	455 (27.5)	18.5
South	593 (35.8)	41.8
West	379 (22.9)	21.0
Political party		
Democrat	818 (49.4)	46.7
Independent, do not know, or none	409 (24.7)	26.8
Republican	429 (25.9)	26.5

Abbreviation: ESS, effective sample size.

^a^ Race and ethnicity were self-reported by study participants. Race and ethnicity were included in this study to assist in describing the sample, which informed the national estimates of Supplemental Nutrition Assistance Program participants’ policy support in 2024 and may be useful in comparing to prior estimates.

^b^ Other category included 70 respondents who identified as non-Hispanic and 2 or more races, 34 who identified as non-Hispanic and Asian or Pacific Islander, and 24 who identified as other, non-Hispanic.

Table 2 displays support for 11 SNAP policies, stratified by respondents’ political party. Overall, 8 policies had majority support: increase SNAP minimum benefit (79.9%; 95% CI, 76.6%-83.1%), increase benefits by 15% (78.8%; 95% CI, 75.5%-82.0%), provide extra funds for healthy foods (74.5%; 95% CI, 71.2%-77.8%), allow prepared foods (72.9%; 95% CI, 69.5%-76.2%), provide extra funds for fruits and vegetables (69.9%; 95% CI, 66.4%-73.4%), increase nutrition education funding (64.9%; 95% CI, 61.4%-68.4%), require SNAP stores to stock healthy items (62.7%; 95% CI, 59.1%-66.3%), and increase SNAP distribution frequency (61.5%; 95% CI, 58.0%-65.1%). Three policies had below-majority support: exclude candy (30.4%; 95% CI, 27.1%-33.7%), exclude sugary drinks (28.9%; 95% CI, 25.8%-32.0%), and prohibit SNAP stores from advertising unhealthy items (24.5%; 95% CI, 21.5%-27.6%). Although support for some policies differed significantly by political party, each policy with majority support overall also had support from most SNAP participants in each party.

**Table 2.** SNAP Policy Support Among All Adult Participants and Stratified by Political Party

SNAP policies	Participants by political party, % (95% CI)				*P* value^a^
	All adults	Democrat	Independent	Republican	
Increase SNAP minimum benefit	79.9 (76.6-83.1)	85.2 (80.9-89.4)	72.0 (65.2-78.7)	78.4 (71.8-85.0)	<.001
Increase benefits by 15%	78.8 (75.5-82.0)	83.2 (78.9-87.5)	70.6 (63.8-77.4)	79.3 (72.6-86.0)	.01
Provide extra funds for healthy foods	74.5 (71.2-77.8)	78.0 (73.5-82.5)	69.2 (62.4-76.0)	73.5 (66.8-80.2)	.10
Allow prepared foods	72.9 (69.5-76.2)	78.2 (73.8-82.6)	61.7 (54.5-69.0)	74.8 (68.3-81.2)	<.001
Provide extra funds for fruits and vegetables	69.9 (66.4-73.4)	74.9 (70.2-79.5)	61.8 (54.7-69.0)	69.3 (62.2-76.4)	.01
Increase nutrition education funding	64.9 (61.4-68.4)	67.6 (62.6-72.5)	65.7 (58.9-72.5)	59.4 (52.3-66.6)	.17
Require SNAP stores to stock healthy items	62.7 (59.1-66.3)	66.7 (61.7-71.6)	57.8 (50.6-65.0)	60.8 (53.4-68.3)	.12
Increase SNAP distribution frequency	61.5 (58.0-65.1)	66.6 (61.4-71.7)	57.4 (50.3-64.5)	56.8 (49.7-63.9)	.04
Exclude candy	30.4 (27.1-33.7)	31.4 (26.6-36.2)	25.8 (19.5-32.1)	33.3 (26.8-39.9)	.23
Exclude sugary drinks	28.9 (25.8-32.0)	29.2 (24.8-33.5)	26.4 (20.0-32.8)	31.0 (24.8-37.1)	.58
Prohibit SNAP stores from advertising unhealthy items	24.5 (21.5-27.6)	26.1 (21.5-30.6)	22.0 (16.2-27.8)	24.4 (18.6-30.2)	.57

Abbreviation: SNAP, Supplemental Nutrition Assistance Program.

^a^ *P* values of policy support differences by political party were calculated using χ^2^ tests. Two-sided *P* < .05 indicated statistical significance.

## Discussion

This nationally representative study found that most SNAP participants, regardless of political affiliation, supported policies to expand SNAP, whereas restrictive policies were broadly unpopular. These findings highlight multiple potentially bipartisan strategies for expanding SNAP that could improve food access and diet quality. For example, similar to past studies, this study found high levels of support for increasing SNAP benefits for healthy foods specifically; evidence suggests increased benefits for healthy foods can improve diet quality and may be cost-effective due to reduced health care spending.^4,5,6^

Study limitations include variation in participants' understanding of policy options and potential nonresponse bias. Although survey weights helped address nonresponse bias, it may still exist due to unobserved differences between respondents and nonrespondents.

As part of Farm Bill discussions, policymakers concerned about SNAP participants’ diet quality may consider prioritizing these policies and other proposals to expand SNAP, given their broad, bipartisan support.

## Notes

Supplement 1. **eAppendix 1.** Survey Administration Details **eAppendix 2.** SNAP Policy Question Wording

Supplement 2.
Data Sharing Statement
