# Supplementary material for: Participant Support for Changes to the Supplemental Nutrition Assistance Program
Source: JAMA Health Forum. 2024 Dec 6;5(12):e244090. doi: 10.1001/jamahealthforum.2024.4090 (PMC11624578; doi:10.1001/jamahealthforum.2024.4090)
Supplement: Supplement 1. — eAppendix 1. Survey Administration Details eAppendix 2. SNAP Policy Question Wording [file jamahealthforum-e244090-s001.pdf]

Supplemental Online Content

Hatton CR, Wolfson JA, Uriarte A, Leung CW. Participant support for changes to the Supplemental Nutrition Assistance Program. *JAMA Health Forum.* Published online December 6, 2024. doi:10.1001/jamahealthforum.2024.4090

eAppendix 1. Survey Administration Details

eAppendix 2. SNAP Policy Question Wording

This supplemental material has been provided by the authors to give readers additional information about their work.

© 2024 Hatton CR et al. *JAMA Health Forum.*

eAppendix 1. Survey Administration Details

AmeriSpeak sampling approach
The AmeriSpeak panel is a probability-based panel administered by NORC at the University of Chicago. Households are randomly selected for participation in the panel using area probability and address-based sampling from the NORC National Sample Frame, which covers 97% of the U.S. household population.

Survey modes
Participants could participate in the survey via internet, telephone, or web access via smartphone.

Estimation of survey weights
The weighting process for this study began with the weights from the AmeriSpeak Panel itself, which are initially estimated using the inverse probability of selection from the NORC National Sample Frame. These weights are then adjusted for both nonresponse and unknown eligibility, and they are raked to population totals from the Current Population Survey and the National Center for Health Statistics for age, gender, census division, race and ethnicity, education, housing tenure, household phone status, age X gender, and age X race and ethnicity.

To estimate the weights for the SNAP Experiences 2024 study, NORC first adjusts the AmeriSpeak panel weights to adjust for unequal probabilities of selection from the panel, nonresponse, and limitations of the sampling frame coverage. In order to produce weights for U.S. adults with incomes below 250% who received SNAP for at least 3 months in 2023, NORC took the following additional steps:

1. Re-rake weights for individuals who completed the screener to be consistent with population estimates for adults with incomes below 250% FPL based on the March 2023 Current Population Survey.
2. Use weighted counts of eligible respondents (those who received SNAP for at least 3 months in 2023) to set benchmarks for the target population.
3. After adjusting for nonresponse, weights are raked to align with population benchmarks using age, gender, census division, race and ethnicity, education, age X gender, age X race and ethnicity, and race and ethnicity X gender.
4. Extreme weights may then be trimmed as needed to prevent large influences in survey estimates and then re-raked to population benchmarks.

Item response rates
33 survey participants did not respond to survey questions about their support for policy changes to SNAP, corresponding to 1.9% of the sample. An additional 4 respondents did not provide their political party, representing .2% of the sample.

© 2024 Hatton CR et al. *JAMA Health Forum.*

eAppendix 2. SNAP Policy Question Wording

How strongly do you support or oppose the following potential policy changes to SNAP? (Strongly oppose, oppose, somewhat oppose, neither support nor oppose, somewhat support, support, strongly support)

1. Increase overall SNAP benefits by 15%
2. Allow purchase of prepared foods (such as a rotisserie chicken) with SNAP benefits
3. Remove sugary drinks (such as soda) from the list of foods that can be purchased with SNAP benefits
4. Remove candy from the list of foods that can be purchased with SNAP benefits
5. Provide extra money to SNAP participants that can only be used to purchase fruits and vegetables
6. Provide extra money to SNAP participants that can only be used to purchase fruits, vegetables, or other healthful foods
7. Increase funding for nutrition education for SNAP participants
8. Require stores that accept SNAP benefits to stock healthy foods and beverages
9. Increase how frequently SNAP benefits are distributed (currently once a month)
10. Prohibit stores that accept SNAP benefits from displaying advertisements for unhealthy foods and beverages
11. Increase the minimum SNAP benefit from $23 to $40 per month

© 2024 Hatton CR et al. *JAMA Health Forum.*
