# Supplementary material for: Participant Support for Changes to the Supplemental Nutrition Assistance Program
Source: JAMA Health Forum. 2024 Dec 6;5(12):e244090. doi: 10.1001/jamahealthforum.2024.4090 (PMC11624578; doi:10.1001/jamahealthforum.2024.4090)
Supplement: Supplement 2. — Data Sharing Statement [file jamahealthforum-e244090-s002.pdf]

# Data Sharing Statement

Hatton. Participant Support for Changes to the Supplemental Nutrition Assistance Program.
*JAMA Health Forum*. Published December 06, 2024. doi:10.1001/jamahealthforum.2024.4090

**Data**
**Data available:** No
